# Cytomegalovirus-specific CD8+ T-cell responses are associated with arterial blood pressure in people living with HIV

**DOI:** 10.1371/journal.pone.0226182

**Published:** 2020-01-13

**Authors:** Vibe Ballegaard, Karin Kaereby Pedersen, Peter Brændstrup, Nikolai Kirkby, Anette Stryhn, Lars P. Ryder, Jan Gerstoft, Susanne Dam Nielsen

**Affiliations:** 1 Department of Infectious Diseases, Viro-immunology Research Unit, Rigshospitalet, University Hospital of Copenhagen, Copenhagen, Denmark; 2 Department of Clinical Immunology, Rigshospitalet, University Hospital of Copenhagen, Copenhagen, Denmark; 3 Department of Immunology and Microbiology, University of Copenhagen, Copenhagen, Denmark; 4 Department of Hematology, Herlev University Hospital, Herlev, Denmark; 5 Department of Medical Microbiology, Rigshospitalet, University Hospital of Copenhagen, Copenhagen, Denmark; Rush University, UNITED STATES

## Abstract

People living with HIV (PLHIV) are at increased risk for cardiovascular disease (CVD), and immunity against cytomegalovirus (CMV) may be a contributing factor. We hypothesized that enhanced T-cell responses against CMV and CMV-IgG antibody-levels are associated with higher arterial blood pressure in PLHIV. We assessed serum CMV-IgG, systolic- (SBP) and diastolic- (DBP) blood pressure, pulse pressure (PP), traditional risk factors, activated CD8+ T-cells (CD38+HLA-DR+), senescent CD8+ T-cells (CD28-CD57+) and interleukin-6 (IL-6) in 60 PLHIV and 31 HIV-uninfected controls matched on age, gender, education and comorbidity. In PLHIV, expression of interleukin-2, tumor necrosis factor-α and interferon-γ was measured by intracellular-cytokine-staining after stimulation of T-cells with CMV-pp65 and CMV-gB. Associations between CMV-specific immune responses and hypertension, SBP, DBP or PP were assessed by multivariate logistic and linear regression models adjusted for appropriate confounders. The median age of PLHIV was 47 years and 90% were male. Prevalence of hypertension in PLHIV was 37% compared to 55% of HIV-uninfected controls. CMV-specific CD8+ T-cell responses were independently associated with higher PP (CMV-pp65; β = 2.29, p = 0.001, CMV-gB; β = 2.42, p = 0.001) in PLHIV. No significant differences were found with regard to individual measures of SBP and DBP. A possible weak association was found between CMV-IgG and hypertension (β = 1.33, p = 0.049) after adjustment for age, smoking and LDL-cholesterol. HIV-related factors, IL-6, CD8+ T-cell activation or CD8+ T-cell senescence did not mediate the associations, and no associations were found between CMV-specific CD4+ T-cell responses and blood pressure in PLHIV. In conclusion, increased arterial blood pressure in PLHIV may be affected by heightened CMV-specific CD8+ T-cell responses.

## Background

Despite treatment with antiretroviral therapy (cART), people living with HIV (PLHIV) have lower life expectancy than HIV-uninfected individuals[1], partly explained by excess risk of cardiovascular diseases (CVD)[2–5]. Hypertension is one of the major CVD risk factors[6], but studies are contradictory as to whether prevalence of hypertension is increased in treated PLHIV compared to uninfected controls [7–10]. However, several studies showed that HIV-related factors such as a low nadir CD4+ T-cell count and longer duration of cART were associated with increased risk of hypertension[8,9,11]. Mechanisms behind excess risk of CVD in PLHIV are multifactorial, and the search for underlying contributing factors is important in order to prevent morbidity and mortality.

CMV is a human β-herpesvirus with a world-wide distribution and a high prevalence in most populations[12,13]. The majority of PLHIV are infected with CMV, and PLHIV have higher T-cell and antibody-specific responses against CMV than HIV-uninfected[14,15]. CMV has been linked to increased risk of CVD-related morbidity and mortality in PLHIV[16], and increased magnitude of CMV-specific immune responses have been associated with subclinical cardiovascular disease[17–19]. The relationship between CMV-specific immune responses and risk of CVD has been thoroughly described in the general population[20–30], where CMV-specific immune responses are associated with risk of hypertension[22,27–30], and CMV-specific T-cells have been shown to have a direct effect on the vascular endothelium[27,31–34]. Thus, the relationship between CMV and adverse CVD-outcomes is not a unique feature of HIV infection, but may be increasingly relevant in this population due to high prevalence of CMV in PLHIV, higher immune responses against CMV, and increasing life expectancy leading to a higher lifetime CMV exposure in PLHIV.

In this study, we hypothesized that higher CMV-specific CD8+ and CD4+ T-cell responses against CMV-pp65 and CMV-gB, or higher serum CMV IgG, would be associated with higher systolic blood pressure, higher pulse pressure and hypertension in PLHIV.

## Methods

### Study population

A total of 60 PLHIV from the outpatient clinic at the Department of Infectious Diseases, Rigshospitalet, Copenhagen, were consecutively included in a study regarding cognitive function and cardiovascular risk profile, and 31 HIV-uninfected controls were selected for comparison and matched overall on age, gender, BMI, education and comorbidity index (assessed by the Charlson comorbidity index). Procedures for recruitment, data collection, demographics, and clinical characteristics of the participants have previously been described in detail[15,35–39]. Nineteen of the controls also participated in a study on diabetes[40]. All PLHIV had suppressed viral replication for ≥ 1 year before inclusion, and had been on stable treatment with cART for ≥2 years. Exclusion criteria were acute illness, chronic infection with hepatitis B virus (HBV) defined by routine measurement of HBsAg/anti-HBc or hepatitis C virus (HCV) defined by routine measurement of anti-HCV/HCV-RNA, intravenous drug use, autoimmune diseases, diabetes (HbA1c > 48 mmol/mol and/or fasting glucose > 7 mmol/L), cancer, or pregnancy. Inclusion and exclusion criteria were selected to eliminate confounders with known impact on non-AIDS comorbidity.

### Ethics approval and consent to participate

The study was approved by the National Committee on Biomedical Research Ethics for the Capital Region of Denmark (H-2-2010-089) and the Danish Data Protection Agency and conducted in accordance with the second declaration of Helsinki. Written informed consent was obtained from all participants.

### Clinical assessments

Detailed information on demographic factors, medical history, smoking, physical activity, medication and data regarding HIV infection were collected at inclusion[35–37]. All examinations were performed by trained medical staff. Waist and hip measurements and body mass index (BMI) calculations were performed[37], and according to WHO guidelines, abdominal obesity was defined as waist-hip ratio ≥ 0.90 for men and ≥ 0.85 for women[41]. Blood pressure (BP) was measured on the left arm after 5 minutes rest and in lying position using the mean of two measurements from a digital blood pressure monitor. Hypertension was defined as anti-hypertensive treatment and/or ≥ 140 mmHg systolic (SBP) and/or ≥ 90 mmHg diastolic (DBP) blood pressure values[42], and pulse pressure (PP) was calculated as the difference between systolic and diastolic blood pressure[43].

### Blood analyses

A fasting venous blood sample was collected and analyzed for total cholesterol, LDL-cholesterol, HDL-cholesterol, HbA1c, and glucose[35,37]. CD4+ T-cell count (cells/uL) was measured as a routine analysis by flow cytometry as previously described[35]. HIV viral load (copies/mL) was measured with a polymerase chain reaction (PCR) quantitative kit (COBAS^®^AmpliPrep/COBAS^®^TaqMan^®^ 48 System; Roche, Basel, Switzerland) according to the manufacturer’s instructions. Nadir CD4 was recorded as the lowest CD4+ T-cell count measured during HIV-infection[35]. As previously described, plasma concentrations of interleukin-6 (IL-6) and tumor necrosis factor-alpha (TNF-α), were measured by Meso-Scale Discovery MULTI-SPOT plate (MSD, Gaithersburg, MD)[35], and T-cell subsets (percentage of activated (CD38+HLA-DR+) and senescent (CD28-CD57+) CD8+ T-cells) were determined using a six-colour FACSCanto flow cytometer (Becton Dickinson, Franklin Lakes, NJ)[35].

### CMV-specific immune responses

In this study we used frozen PBMC´s from the participants as a source of both antigen presenters and T-cells and short term stimulation (6 hours) with 15mer peptides. The method is a standardized and validated approach that has been used by multiple groups to determine ex vivo T-cell responses[44–48]. The method has been described in detail in a previous publication[15]. In CMV-seropositive PLHIV (n = 54), CMV-specific CD8+ and CD4+ T-cell responses were determined by measurement of intracellular expression of IL-2, TNF-α and IFN-γ after stimulation of peripheral blood mononuclear cells (PBMC) with CMV-pp65 and CMV-gB[15]. We used the CFC Becton Dickinson assay that has been optimized by the manufacturer and with a recommended stimulation time of 6 hours (BD Bioscience)[46]. In brief, thawed PBMC were stimulated in duplicate samples using either a CMV-pp65 peptide pool containing 138 peptides derived from a peptide scan through 65 kDa phosphoprotein (pp65) (Swiss-Prot ID: P06725), a CMV-gB peptide pool of 224 peptides derived from a peptide scan through Envelope glycoprotein B (gB) (Swiss-Prot ID: P06473), or Staphylococcal enterotoxin B (SEB) (2.5 ug/mL; Sigma-Aldrich) as a positive control. The stimulated PBMC were incubated in the presence of co-stimulatory anti-CD28/CD49d (BD Biosciences) for six hours at 37ºC and Brefeldin A (BD Biosciences) was added after 2 hours. An unstimulated control was incubated with DMSO, anti-CD28/CD49d and Brefeldin A in order to detect background staining. Flourescence Minus One (FMO) controls were used as gating controls, and isotype controls were used to check for background due to nonspecific antibody binding.

At the end of stimulation, cells were washed, stained with BD Horizon^™^ Fixable Viability Stain 450 (FVS450), treated with FACS lysing- and FACS permeabilization solution (BD FACS^™^) and stained with anti-CD4-FITC/anti-CD69-PE/anti-CD3-PerCP (clone SK3/L78/SK7 BD fastimmune^™^), anti-CD8-V500 (clone SK1 BD Horizon^™^), anti-IL-2-BV421 (clone 5344.111 BD Horizon^™^), anti-TNF-α -APC (clone 6401.1111 BD FastImmune^™^) and anti-IFN-γ-PE-Cy7 (clone B27 BD Pharmingen^™^), at 22ºC for 30 minutes. Cytokine responses were acquired immediately using a BD FACSCanto^™^ II flow cytometer and flow cytometry results were analysed using BD FACSDiva (v8.0.1) software (BD Biosciences). The gating strategy has previously been described[15]. In brief, a lymphocyte gate based on FSC/SSC, a singlet gate, and a live/dead cell gate were applied before gating on CD3+CD4+ and CD3+CD8+ cells. Further, for each T-cell subset, CD69+ populations were gated from CD69+ histograms for CD4+ and CD8+ populations, and expression of IFN-γ, TNF-α and IL-2 was then determined from the CD4+ and CD8+ populations. Expression of IFN-γ, TNF-α and IL-2 was determined from the CD4+CD69+ and CD8+CD69+ populations, and co-expression patterns were analyzed by application of a combinational gating strategy. Net subset frequencies of CMV-specific CD4+ and CD8+ T-cells were determined after background subtraction, and a positive cytokine response was defined as above 0.01% of the reference subset (CD4+ or CD8+ T-cells) or at least 40 events[49,50]. By summing up the frequency of CD4+ or CD8+ T-cells within each unique combination of functions (IFN-γ, TNF-α or IL-2), we analyzed the magnitude of the total CMV-specific response (%CD8+ or %CD4+). Thus, each responding cell was calculated only once.

CMV IgG antibody levels were measured in participants with available frozen serum samples (n = 85), using a commercial chemiluminescent immunoassay (LIAISON ^®^ CMV IgG II, DiaSorin S.P.A., Saluggia, Italy)[51], and antigen-binding avidity of CMV IgG antibodies in serum was measured with the LIAISON^®^ CMV Avidity II assay (DiaSorin S.P.A., Saluggia, Italy)[52], according to manufacturer’s instructions. Plasma CMV DNAemia was assessed using the Amplicor CMV Monitor test (Roche Diagnostics, Indianapolis, IN).

### Statistical analysis

Continuous variables were reported as median and interquartile range, while categorical data as percentage of total number and frequency. Different groups were compared with Student t-test or Mann Whitney test for continuous variables or Χ^2^-test/Fisher´s tests for categorical variables, where appropriate. Univariate and multivariable logistic models were used to test the association between CMV-specific T-cell responses and CMV-IgG and hypertension. Unadjusted and adjusted odds ratios (OR/aOR) and 95% confidence intervals [CIs] were reported. In addition, linear regression analysis was performed with CMV-IgG or CMV-specific CD4+ and CD8+ T-cells as independent variables and continuous measures of SBP, DBP and PP as dependent variables. Covariates were selected based on clinical assumptions or known associations with increased arterial blood pressure (age, education, sex, LDL-cholesterol, smoking, HbA1c, abdominal obesity, CMV IgG and HIV status). Due to a small sample size and few events, a backward elimination selection approach including only covariates with p<0.20 in univariate analysis and α = 0.10 was applied to build a basic multivariate model including CMV-IgG (per 100 U/ml)/total CMV-specific T-cell responses (% of CD4+ or CD8+ T-cells) in addition to age (per decade), LDL-cholesterol (per mmol/L) and smoking (yes/no). Using the same backward elimination selection approach, all the basic multivariate models included adjustment for age, smoking and LDL-cholesterol. Separate multivariate models for PLHIV were created to assess the association between CMV-specific immune responses and hypertension, SBP, DBP or PP while considering selected HIV-related (nadir CD4+, CD4+/CD8+ ratio and duration of HIV infection) or immunologic confounders (IL-6, senescent CD8+ T-cells, activated CD8+ T-cells). Selected covariates were added to the multivariate models one by one. Additional models were only created when the association was positive in the basic multivariate models. Residuals were checked for normal distribution and log transformation was performed when appropriate. False discovery rate (Benjamini-Hochberg method) adjusted p-values were calculated to adjust for multiple testing. A P-value of <0.05 was considered statistically significant, and statistical analysis was performed using SAS (version 9.4 SAS Institute, Copenhagen, Denmark) or GraphPad Prism 7 (GraphPad Software Inc).

## Results

### Study participants

A total of 60 PLHIV and 31 HIV-uninfected controls were included. Demographic and clinical characteristics are shown in summary in Table 1, and have previously been published[15,35–39].

**Table 1:** General characteristics of the study population.

	PLHIV n = 60	HIV-uninfected controls n = 31	p
Age, years, median, (IQR)	47 (44–54)	50 (42–55)	0.936
Gender, male, % (n)	89.7 (52)	83.9 (26)	0.505
CMV positive, % (n)	91.5 (54)	64.3 (18)	0.004
**Blood pressure**			
Hypertension, % (n)	37.3 (22)	54.8 (17)	0.110
Anti-hypertensive treatment, % (n)	6.8 (4)	6.5 (2)	0.953
SBP, mmHg, median (IQR)	133 (122–144)	136 (128–146)	0.426
DBP, mmHg, median (IQR)	80 (75–87)	87 (76–92)	0.201
PP, mmHg, median (IQR)	50 (43–57)	53 (46–57)	0.952
**Cardiovascular risk factors**			
Current smokers, % (n)	35.6 (21)	12.9 (4)	0.022
LDL-C, mmol/l, median (IQR)	3.5 (2.6–3.8)	3.1 (2.6–3.5)	0.340
HbA1c, mmol/mol, median (IQR)	5.3 (5.1–5.6)	5.4 (5.2–5.6)	0.134
Abdominal obesity, % (n)	20.7 (12)	41.9 (13)	0.034
BMI, median (IQR)	23.4 (21.5–24.4)	24.9 (23.7–26.0)	0.011
**CMV-specific immune response in CMV seropositive PLHIV (n = 54) and HIV-uninfected (n = 18)**			
CMV IgG levels, U/ml, median (IQR)	215 (119–532)	111 (79–132)	0.001
CMV-specific pp65 CD4+ T-cells, % of CD4+	1.1 (0.5–2.2)	-	-
CMV-specific pp65 CD8+ T-cells, % of CD8+	1.6 (0.7–3.9)	-	-
CMV-specific gB CD4+ T-cells, % of CD4+	1.0 (0.4–1.4)	-	-
CMV-specific gB CD8+ T-cells, % of CD8+	1.6 (0.6–3.9)	-	-
**HIV-specific variables**			
CD4+, median (IQR)	570 (420–720)	-	-
CD4+ nadir, median (IQR)	150 (58–260)	-	-
CD4+ nadir < 200, % (n)	57.6 (34)	-	-
CD4+/CD8+ ratio, median (IQR)	0.8 (0.5–1.1)	-	-
HIV duration, years, median (IQR)	9 (6–13)	-	-

Demographic and clinical characteristics from this cohort has previously been published[15,35–39]. Abbreviations: BMI; body mass index, CMV; cytomegalovirus, DBP; diastolic blood pressure, HbA1c; glycated hemoglobin, LDL-C; low density lipoprotein cholesterol, n; number, PLHIV; people living with HIV, PP; pulse pressure, IQR; interquantile range, SBP; systolic blood pressure.

Current CMV reactivation or primary CMV infection was not detected, since none of the participants had detectable CMV DNA in plasma, and all had an avidity index above 0.20. In addition, all PLHIV had suppressed HIV-RNA. In PLHIV 37% had hypertension compared to 57% of HIV-uninfected controls. However a significant difference in risk of hypertension could not be identified, and no significant differences were detected in SBP, DBP or PP (Table 1). In addition, HIV infection was not associated with increased risk of hypertension in univariate analysis or when adjusting for age, LDL-cholesterol and smoking (aOR 0.51 (0.19–1.39), p = 0.191).

### Increased CMV-specific CD8+ T-cell responses were associated with higher pulse pressure in PLHIV

**Fig 1:**
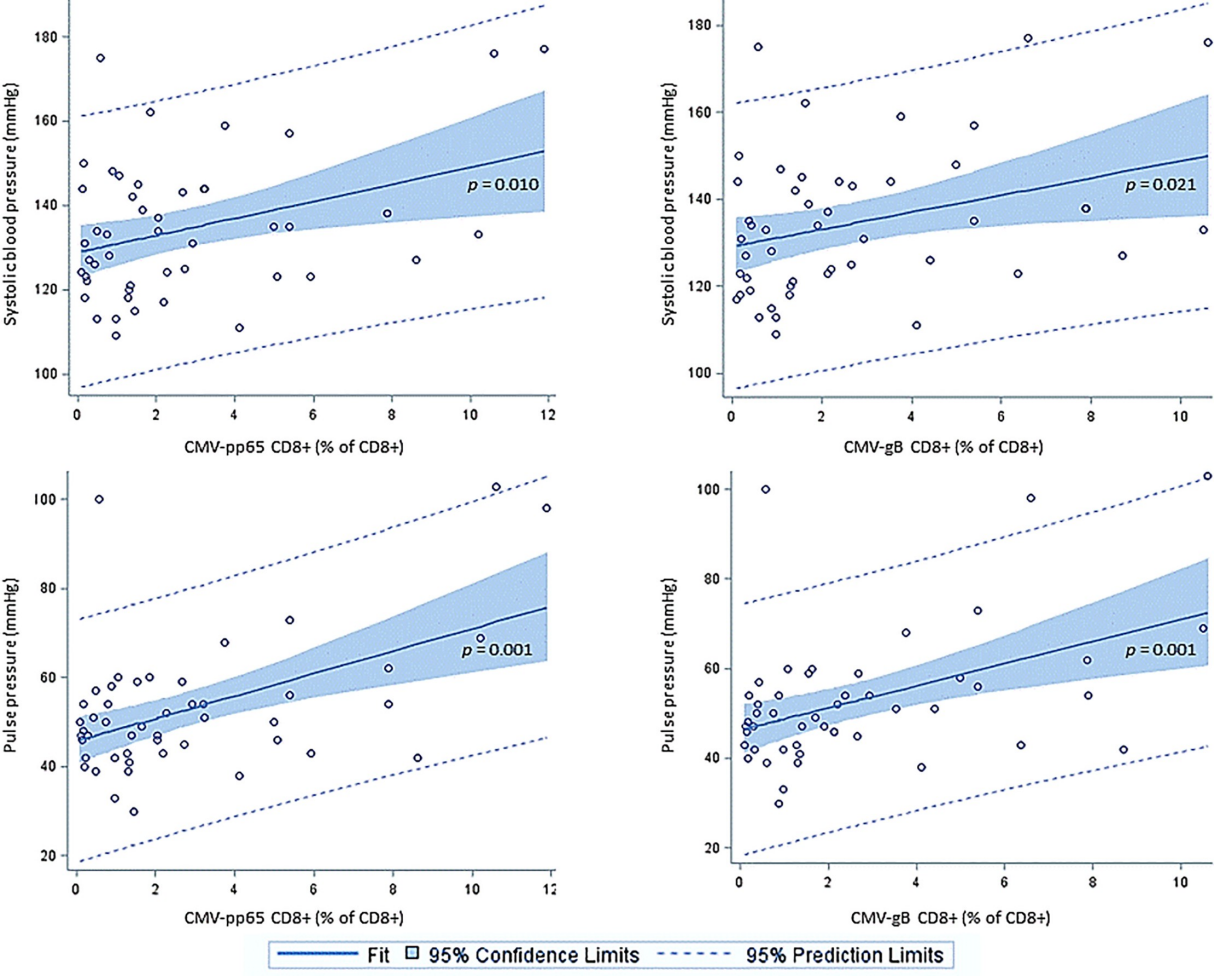
CMV-specific CD8+ T-cell responses are associated with systolic blood pressure and pulse pressure in PLHIV. Simple linear regression with CMV-specific (pp65 and gB) CD8+ T-cells (% of CD8+) as independent variable and systolic blood pressure or pulse pressure as dependent variables. A fitted plot with 95% confidence limits is shown.

In CMV-seropositive PLHIV, there was a consistent association between increased CMV-specific CD8+ T-cell responses and PP in univariate analysis and also in multivariate analysis, after adjustment for age, smoking and LDL-cholesterol (Table 2, Fig 1). We could not consistently confirm, that the same association was present between increased CMV-specific CD8+ T-cell responses (% of CD8+ T-cells) and higher SBP (mmHg). (Table 2, Fig 1). No associations were found between CMV-specific CD8+ T-cells and DBP, and higher CMV-specific CD8+ T-cell responses were not associated with hypertension in PLHIV (Table 2). In addition, we did not find that CMV-specific CD4+ T-cell responses were associated with SBP, PP, or hypertension in PLHIV (S1 Table).

**Table 2:** Univariate and multivariate regression models analysing associations between CMV-specific CD8+ T-cell responses and blood pressure in PLHIV.

	Univariate model		Basic multivariate model adjusted for age, smoking and LDL-cholesterol		Additional multivariate models adjusted for age, smoking and additional covariates one at a time:							
**Hypertension**	OR (95% CI)	P	aOR (95% CI)	p	Nadir CD4+		CD4+/CD8+		HIV duration		Senescent CD8+	
CMV-pp65-CD8+	1.15 (0.95–1.41)	0.158	1.24 (0.95–1.62)	0.112	-	-	-	-	-	-	-	-
CMV-gB-CD8+	1.20 (0.96–1.49)	0.107	1.09 (0.97–1.23)	0.163	-	-	-	-	-	-	-	-
**Systolic BP**	**β (95% CI)**	**P**	**β (95% CI)**	**p**	**β**	**P**	**β**	**P**	**β**	**P**	**β**	**P**
CMV-pp65-CD8+	2.01 (0.50–3.52)	0.010	1.57 (0.12–3.01)	0.035	1.63	0.036	1.80	0.025	1.60	0.042	1.60	0.045
CMV-gB-CD8+	1.97 (0.31–3.63)	0.021	1.75 (0.23–3.27)	0.025	1.59	0.068	1.83	0.040	1.58	0.062	1.56	0.071
**Diastolic BP**	**β (95% CI)**	**p**	**β (95% CI)**	**p**								
CMV-pp65-CD8+	-0.52 (-1.32–0.29)	0.202	-0.72 (-1.51–0.08)	0.077	-	-	-	-	-	-	-	-
CMV-gB-CD8+	-0.51 (-1.40–0.31)	0.255	-0.64 (-1.51–0.23)	0.143	-	-	-	-	-	-	-	-
**Pulse pressure**	**β (95% CI)**	**p**	**β (95% CI)**	**p**	**β**	**P**	**β**	**P**	**β**	**P**	**β**	**P**
CMV-pp65-CD8+	2.51 (1.24–3.78)	0.001	2.44 (1.15–3.74)	0.001	2.42	0.001	2.62	0.001	2.46	0.001	2.33	0.002
CMV-gB-CD8+	2.44 (1.00–3.89)	0.001	2.42 (1.05–3.80)	0.001	2.34	0.003	2.61	0.001	2.33	0.003	2.30	0.004

Multivariate models with CMV-specific CD8+ T-cell responses (% of CD8+ T-cells) as the independent variable and blood pressure as outcome variable. Logistic regression models were used when hypertension was the outcome variable, and linear regression models were used when systolic blood pressure (mmHg), diastolic blood pressure (mmHg), or pulse pressure (mmHg) were the outcome variables. A backward elimination selection approach including only covariates with p<0.20 in univariate analysis and α = 0.10 was used. The covariates (age, education, sex, LDL-cholesterol, smoking, HbA1c and abdominal obesity) were selected based on clinical assumptions. The basic multivariate models were minimized to include age, smoking and LDL-cholesterol. Separate multivariate models were created to evaluate the effect of additional covariates. Each model included age, smoking, and additional covariates included one at a time (nadir CD4+, CD4+/CD8+, duration of HIV, plasma IL-6, activated CD8+ T-cells (% of CD8+) and senescent CD8+ T-cells (% of CD8+). IL-6 and activated CD8+ T-cells did not alter the associations and is not shown in the table.

False discovery rate adjusted p-value: total CMV-pp65 CD8+ T-cells responses versus Systolic BP: p = 0,027., total CMV-gB CD8+ T-cell responses versus Systolic BP: p = 0,042., total CMV-pp65 CD8+ T-cells responses versus Pulse Pressure: p = 0.004, total CMV-gB CD8+ T-cell responses versus Pulse Pressure: p = 0.004. Abbreviations: aOR; adjusted odds-ratio, CMV; cytomegalovirus, CI; confidence interval, OR; odds-ratio, p; p-value.

### Impact of HIV-related factors on associations between CMV-specific CD8+ T-cell responses and pulse pressure in PLHIV

Nadir CD4+, duration of HIV infection and the CD4+/CD8+ ratio was added to the multivariate models one at a time, to determine whether these HIV-related factors might mediate the association between CMV-specific CD8+ T-cell responses and pulse pressure in PLHIV. The associations between CMV-specific CD8+ T-cells and PP were not affected by adjustment for HIV-related factors (Table 2). In addition, we tested whether adjusting for HIV-related factors would change the association between CMV-specific CD8+ T-cells responses and systolic blood pressure, and we did not find that these factors consistently affected the associations.

### Impact of T-cell activation, T-cell senescence, and IL-6 on associations between CMV-specific CD8+ T-cell responses and pulse pressure in PLHIV

CD8+ T-cell activation (CD38+HLA-DR+, % of CD8+), CD8+ T-cell senescence (CD28-CD57+, % of CD8+), and plasma IL-6 (pg/mL) was added to the basic multivariate models one at a time to determine the impact of immunologic factors often associated with both CMV, HIV and vascular aging.

None of the immunologic factors had an impact on the associations between CMV-specific CD8+ T-cell responses and PP (data shown in Table 2 and in S2 Table).

### Serum CMV IgG levels and hypertension in PLHIV

In PLHIV, no associations were found between CMV IgG and hypertension in univariate analysis, but a possible weak association was found after adjustment for age, smoking and LDL-cholesterol (Table 3). When investigating continuous measures of blood pressure, CMV IgG was not associated with elevated SBP, DBP or PP, in either univariate or multivariate linear regression analysis (data shown in S3 Table).

**Table 3:** Univariate and multivariate logistic regression analysis investigating risk factors for hypertension in CMV-seropositive PLHIV.

**Hypertension**	PLHIV (n = 54)			
	OR (95% CI)	P	aOR (95% CI)	P
**CMV IgG**	1.11 (0.89–1.38)	0.356	1.33 (1.01–1.77)	0.049
**per 100 U/ml**				
**Age**	2.03 (0.96–4.32)	0.065	2.16 (0.90–5.19)	0.084
**per 10 years**				
**Smoking**	2.59 (0.79–8.52)	0.117	7.47 (1.46–38.31)	0.016
**yes vs. no**				
**LDL**	1.82 (0.95–3.48	0.072	2.51 (1.14–5.54)	0.023
**per mmol/L**				

CMV-seropositive PLHIV (n = 54) were included. Covariates were selected based on clinical assumptions or known associations with hypertension (age, education, sex, LDL-cholesterol, smoking, HbA1c, abdominal obesity, CMV IgG and HIV status). Due to a small sample size, a backward elimination selection approach including only covariates with p<0.20 in univariate analysis and α = 0.10 was applied to build a basic multivariate model including CMV-IgG (per 100 U/ml), age (per decade), LDL-cholesterol (per mmol/L) and smoking (yes/no). Abbreviations: aOR; adjusted odds-ratio, CMV; cytomegalovirus, CI; confidence interval, OR; odds-ratio, p; p-value, PLHIV; people living with HIV

## Discussion

This study identified a possible relationship between CMV-specific cellular immunity and blood pressure in PLHIV on stable treatment. Increased CMV-specific CD8+ T-cell responses were independently associated with higher pulse pressure.

In the present study, 37% of PLHIV had hypertension compared to 57% of HIV-uninfected controls, but a significant difference in risk of hypertension was not identified which may be due to a small sample size and risk of type II errors. Importantly, the control group was matched on age, gender, education and comorbidity index[35]. For comparison, the prevalence of hypertension was 44% among PLHIV and 58% among controls in a recent large Danish cohort study of predominantly well-treated PLHIV[8]. In that study, HIV was associated with a lower risk of hypertension[8]. Although prevalence of hypertension may not be increased in PLHIV, the causal factors leading to hypertension may still differ, and it is important to understand the pathogenesis in order to prevent CVD and CVD-related mortality.

CMV-specific T-cell responses are elevated in PLHIV compared to HIV-uninfected controls[14,17,53], and CMV-specific CD8+ T-cell responses have been associated with carotid intima media thickness (cIMT)[17]. In addition, studies in HIV-uninfected populations has provided evidence of a relationship between CMV-specific immune responses and elevated blood pressure or arterial stiffness[22,27–30]. However, no previous studies have investigated the relationship between blood pressure and CMV-specific T-cell immunity in PLHIV. In line with previous studies, we did not find associations between CMV-specific CD4+ T-cells and blood pressure. Although the CD4+ and CD8+ T-cell responses are linked, it is a well-known feature of CMV immunity in both PLHIV and HIV-uninfected, that the CMV-specific CD8+ T-cell compartment is expanded in comparence to the CD4+ T-cell compartment[14,44,45] but we were not able to further address the mechanism behind these observations.

In PLHIV, a recent study showed that PLHIV on cART had increased arterial stiffness compared to a matched control group[54]. Arterial stiffness is a strong predictor of CVD and CVD-related mortality[55]. Non-invasive surrogate markers of arterial stiffness are typically pulse pressure and isolated systolic hypertension[56], and some studies indicate that PP may better characterize arterial stiffness than SBP[43,57].Traditional risk factors may play a role, but in this study the association between CMV-specific CD8+ T-cell responses and pulse pressure, was still present after adjustment for traditional risk factors.

The mechanism by which CMV-specific CD8+ T-cells contribute to elevated blood pressure or arterial stiffness has not been identified. We previously showed that CMV-specific CD8+ T-cell responses were associated with senescence and terminal differentiation of CD8+ T-cells in treated PLHIV[15]. In addition, senescent CD8+ T-cells has been associated with arterial stiffness and hypertension in HIV-uninfected individuals[30,58], and increased frequencies of activated and senescent CD8+ T-cells were associated with prevalence of carotid artery lesions and arterial stiffness in PLHIV[59–61]. The action of activated and/or senescent CD8+ T-cells on the endothelium could be direct (as in cytotoxicity) or indirect through increased release of proinflammatory cytokines. In the present study, activated or senescent CD8+ T-cells and/or serum levels of IL-6 was not associated with elevated blood pressure, and did not confound the association between CMV-specific CD8+ T-cell responses and pulse pressure. Since our study is observational and not mechanistic, we are not able to further address the mechanism behind the observed associations.

Duration of HIV infection and biological age are important confounders to consider. The majority of PLHIV are CMV-seropositive and the CMV-specific memory T-cell compartment is inflated during aging. This could lead to CMV immunity being randomly associated with hypertension. We address this by adjusting for age in the basic multivariable models, and adjustment for duration of HIV infection is included in additional multivariate models. The associations were stable when adjusting for age and duration of HIV infection. In a previous study, we found that nadir CD4+ and duration of HIV infection was associated with CMV IgG, but not with CMV-specific T-cell responses[15], and a low nadir CD4+ has been associated with increased risk of hypertension in several studies[8,11,62]. We hypothesized that the effect of CMV immunity on blood pressure was mediated by effects of late stage HIV defined by a low nadir CD4+ or inversed CD4+/CD8+ ratio. However, the association between CMV-specific CD8+ T-cells and PP was not affected, and we did not find support of this hypothesis. However, importance of HIV-related factors should be addressed in future studies.

CMV IgG has been established as a cardiovascular risk factor [29] [20,21,23,25], and has often been interpreted as a surrogate marker of subclinical CMV reactivations. However, CMV-IgG probably reflect a combined effect of lifetime burden of CMV, recent reactivations and reinfections, and host factors determining ability to suppress CMV replication[63,64].

PLHIV in our study had higher levels of CMV IgG than HIV-uninfected controls, and a possible weak association was found between higher CMV IgG and hypertension after adjustment for age, LDL-cholesterol and smoking. Because our sample size was small and number of events was limited the finding is exploratory. However, previous studies suggest that increased CMV IgG may be related to subclinical CVD in PLHIV[18,19] and also in HIV-uninfected populations,

The present study has limitations. First, it is a cross-sectional study with inclusion of a selected and limited number of participants. Therefore, direct cause-effect relationships could not be identified, and results may not be applicable to the general HIV population. Secondly, the small sample size increases the risk of type II errors, and limits the possibility to adjust for all appropriate confounders when multivariate models are created. Thus, the results should be interpreted with caution and should be confirmed in studies with larger sample sizes. Additionally, this study is descriptive and mechanistic studies are required to address mechanisms by which CMV-specific CD8+ T-cells contribute to increased blood pressure or arterial stiffness in treated PLHIV. Nearly all PLHIV in our cohort were CMV-seropositive, so we were not able to compare CMV-seropositive and CMV-seronegative individuals, although such comparisons would have added important extra information to the study. In addition, we were not able to perform analysis of CMV-specific T-cell responses in the control group due to insufficient amounts of PBMC although such analyses would have added relevant information to the study.

Since more than 150 CMV epitopes are immunogenic for CD8+ and CD4+ T-cells, and stimulation with 19 specific CMV epitopes are required to estimate the total CD8+ and CD4+ T-cell response to CMV[45], future studies would benefit from a full characterization of CMV-specific cellular immunity[65].

In addition, since CMV-specific CD8+ T-cell responses are polyclonal and major variations between individuals in response to CMV-antigens has been demonstrated[66], it is a limitation that we were not able to use a broader range of CMV-specific antigens in the assay. The various antigen-specific CD8+ T-cells could additively or synergistically affect blood pressure, and a broader representation of CMV antigens might have found stronger associations than presented in this study.

A strength of the study is a well-characterized cohort in terms of immunologic, cardiovascular and metabolic risk factors and important confounders were excluded since we used strict inclusion criteria. The precise role of CMV-specific immune responses with regard to hypertension and premature vascular aging in HIV infection warrants further investigations.

## Supporting information

S1 Table Univariate and multivariate logistic regression investigating associations between CMV-specific CD4+ T-cell responses and blood pressure in people living with HIV. (PDF)

S2 Table Multivariate linear regression analysis investigating associations between CMV-specific CD8+ T-cells and pulse pressure in PLHIV while adjusting for immunologic factors (IL-6, senescent CD8+ T-cells and activated CD8+ T-cells). (PDF)

S3 Table Univariate and multivariate linear regression investigating associations between CMV-IgG and systolic blood pressure, diastolic blood pressure and pulse pressure in people living with HIV. (PDF)

## Abbreviations

BMI: Body mass index
BP: blood pressure
cART: Highly active antiretroviral therapy
cIMT: carotid intima media thickness
CMV: Cytomegalovirus
CVD: Cardiovascular disease
CX3CR1: Fractalkine-CX3C motif chemokine receptor 1
DBP: Diastolic blood pressure
HBV: Hepatitis B virus
HCV: hepatitis C virus
HIV: Human immunodeficiency virus
ICS: intracellular cytokine staining assay
IgG: Immunoglobulin G
IL: Interleukin
PBMC: peripheral blood mononuclear cells
PLHIV: People living with HIV
PP: Pulse pressure
SBP: Systolic blood pressure
SEB: Staphylococcal enterotoxin B
TNF: tumor necrosis factor
