# Supplementary material for: Cytomegalovirus-specific CD8+ T-cell responses are associated with arterial blood pressure in people living with HIV
Source: PLoS One. 2020 Jan 13;15(1):e0226182. doi: 10.1371/journal.pone.0226182 (PMC6957152; doi:10.1371/journal.pone.0226182)
Supplement: S1 Table — (PDF) [file pone.0226182.s001.pdf]

Table 1. Univariate and multivariate logistic regression investigating associations between CMV-specific CD4+ T-cell responses and blood pressure in people living with HIV

**Hypertension**	**Univariate OR (95 % CI)**	**p**	**Multivariate aOR (95 % CI)**	**p**
CMV-pp65-CD4, % of CD4+	0.99 (0.74-1.32)	0.945	0.99 (0.70-1.39)	0.937
CMV-gB-CD4, % of CD4+	1.03 (0.67-1.60)	0.884	1.07 (0.64-1.77)	0.801
**Systolic blood pressure**	**β (95 % CI)**	**p**	**Adjusted β (95 % CI)**	**p**
CMV-pp65-CD4, % of CD4+	-0.65 (-3.13-1.82)	0.597	-0.61 (-3.14-1.93)	0.630
CMV-gB-CD4, % of CD4+	-0.91 (-4.69-2.87)	0.628	-0.73 (-4.72-3.26)	0.712
**Pulse pressure**	**β (95 % CI)**	**p**	**Adjusted β (95 % CI)**	**p**
CMV-pp65-CD4, % of CD4+	-0.70 (-2.97-1.57)	0.538	-0.55 (-2.96-1.86)	0.648
CMV-gB-CD4, % of CD4+	-2.00 (-7.05-3.05)	0.428	-0.69 (-4.50-3.13)	0.718

Multivariate models adjusted for age, smoking, and LDL-cholesterol, and additional HIV-associated variables in separate multivariate models
