# Supplementary material for: Cytomegalovirus-specific CD8+ T-cell responses are associated with arterial blood pressure in people living with HIV
Source: PLoS One. 2020 Jan 13;15(1):e0226182. doi: 10.1371/journal.pone.0226182 (PMC6957152; doi:10.1371/journal.pone.0226182)
Supplement: S2 Table — (PDF) [file pone.0226182.s002.pdf]

Table 2. Multivariate linear regression analysis investigating associations between CMV-specific CD8+ T-cells and pulse pressure in PLHIV while adjusting for immunologic factors (IL-6, senescent CD8+ T-cells and activated CD8+ T-cells)

**CMV-pp65 CD8+ T-cells**	**B (95% CI)**	**p**
IL-6 (pg/ml)	2.56 (1.28-3.84)	0.002
Activated CD8+ T-cells (% of CD8+)	2.43 (1.09-3.77)	0.001
**CMV-gB CD8+ T-cells**	**B (95% CI)**	**p**
IL-6 (pg/ml)	2.39 (1.01-3.80)	0.002
Activated CD8+ T-cells (% of CD8+)	2.45 (1.04-3.94)	0.002

Linear regression models with pulse pressure as outcome variable and CMV-specific CD8+ T-cells as predictor variable. Models are adjusted for age, smoking, and one additional variable. Variables in linear regression analysis were log-transformed when appropriate.
