# Supplementary material for: Cytomegalovirus-specific CD8+ T-cell responses are associated with arterial blood pressure in people living with HIV
Source: PLoS One. 2020 Jan 13;15(1):e0226182. doi: 10.1371/journal.pone.0226182 (PMC6957152; doi:10.1371/journal.pone.0226182)
Supplement: S3 Table — (PDF) [file pone.0226182.s003.pdf]

Table 3. Univariate and multivariate linear regression investigating associations between CMV-IgG and systolic blood pressure, diastolic blood pressure and pulse pressure in people living with HIV

**Systolic blood pressure**	**Unadjusted β (95 % CI)**	**P**	**Adjusted β (95 % CI)**	**p**
CMV IgG, 100 U/ml	-0.14 (-2.13-1.84)	0.885	0.33 (-1.65-2.30)	0.742
**Diastolic blood pressure**	**Unadjusted β (95 % CI)**	**p**	**Adjusted β (95 % CI)**	**p**
CMV IgG, 100 U/ml	0.09 (-0.97-1.15)	0.867	0.16 (-0.96-1.28)	0.779
**Pulse pressure**	**Unadjusted β (95 % CI)**	**p**	**Adjusted β (95 % CI)**	**p**
CMV IgG, 100 U/ml	-0.23 (-2.00-1.53)	0.792	0.17 (-1.70-2.03)	0.857
